# SERS Detection of Biomolecules by Highly Sensitive and Reproducible Raman-Enhancing Nanoparticle Array

**DOI:** 10.1186/s11671-017-2121-x

**Published:** 2017-05-10

**Authors:** Tzu-Yi Chan, Ting-Yu Liu, Kuan-Syun Wang, Kun-Tong Tsai, Zhi-Xin Chen, Yu-Chi Chang, Yi-Qun Tseng, Chih-Hao Wang, Juen-Kai Wang, Yuh-Lin Wang

**Affiliations:** 1 Department of Materials Engineering, Ming Chi University of Technology, New Taipei City, 24301 Taiwan; 2 Institute of Atomic and Molecular Sciences, Academia Sinica, Taipei, 10617 Taiwan; 3 Center for Condensed Matter Sciences, National Taiwan University, Taipei, 10617 Taiwan; 4 Department of Physics, National Taiwan University, Taipei, 10617 Taiwan

**Keywords:** Surface-enhanced Raman scattering (SERS), Nanoparticle arrays, Biomolecules detection

## Abstract

**Abstract:**

This paper describes the preparation of nanoarrays composed of silver nanoparticles (AgNPs: 20–50 nm) for use as surface-enhanced Raman scattering (SERS) substrates. The AgNPs were grown on porous anodic aluminum oxide (AAO) templates by electrochemical plating, and the inter-channel gap of AAO channels is between 10 and 20 nm. The size and interparticle gap of silver particles were adjusted in order to achieve optimal SERS signals and characterized by scanning electron microscopy, atomic force microscopy, and Raman spectroscopy. The fluctuation of SERS intensity is about 10–20% when measuring adenine solutions, showing a great reproducible SERS sensing. The nanoparticle arrays offer a large potential for practical applications as shown by the SERS-based quantitative detection and differentiation of adenine (A), thymine (T), cytosine (C), guanine (G), β-carotene, and malachite green. The respective detection limits are <1 ppb for adenine and <0.63 ppm for β-carotene and malachite green, respectively.

**Graphical Abstract:**

Uniform and reproducible Raman enhancement enabled by Ag nanoparticle array embedded in anodic aluminum oxide differentiates and helps quantify DNA canonical nucleobases (adenine, thymine, cytosine, and guanine).

## Background

Surface-enhanced Raman spectroscopy (SERS) nanotechnology is an interesting platform for rapid and precise identification of small biomolecules and becomes a potentially fingerprinting and bio-detecting technology, due to enhance Raman signals by 6–13 orders of magnitude in the SERS-active surface [1–11]. The SERS-active surface was fabricated by the arrangement of silver or gold nanoparticle arrays, which generated the localized surface plasmon resonances and the laser exposure in the analytic biomolecules.

The key point of SERS technology is focused on controlling the interparticle gap and the diameter of the metal nanoparticles. The report demonstrated that “hot-spot” effect can be generated when the interparticle gap is lower than 10 nm, which will enormously increase SERS signals when the analytic biomolecules are close to the SERS-active surface. However, the hot-spot effect of the SERS-active substrate is immensely enhanced with decreased interparticle gap-to-particle diameter ratio, which can affect the stability of SERS activity if the variation of the interparticle gap and the particle diameter cannot be brought into control. The detection of this SERS-active substrate has achieved monolayer sensitivity, which is useful to detect a small number of biomolecules observed in a cellular compartment [12, 13], such as sensing in immunoassays, DNA, cancer cells, and microbes [4, 14–20].

In our previous study of Ag nanoparticles embedded in ordered array of anodic aluminum oxide (AgNP/AAO substrate) [21], scattering spectra-rather than transmission or reflection spectra owing to their optical inference-of such array with different interparticle gaps were acquired to reveal their electromagnetic resonance properties. Analytic formulae were derived based on electrostatic dipole approximation to describe the resonance wavelength and width as a function of dimensional factors of the Ag nanoparticle array (particle diameter and interparticle spacing). The experimental results are in good agreement with the derived formulate. For SERS applications, since the localized surface plasmon resonance (LSPR) wavelength in the present work (interparticle spacing: 10–30 nm) is in the range of 500–700 nm (peaked at 620 nm), He–Ne laser (632.8 nm) was used as the excitation light source to boost the local excitation field and the ensuing emission propensity. Furthermore, for simulation study, we performed high precision electrodynamic simulation based on pseudo-spectral time-domain (PSTD) method in our previous study [22]. The far-field scattering spectra thus obtained from calculation also showed good agreement with experimental findings. The surface electric and magnetic fields of the Ag nanoparticle array under two polarization excitation schemes (along *x*- and *y*-axis) were calculated. The enhanced electric local field was manifested at the gap between adjacent nanoparticles. 

The AgNP/AAO substrate was investigated with scanning electron microscopy (SEM) and atomic force microscopy (AFM).  Its SERS sensitivity and reproducibility are presented in this report. Finally, the SERS detection of small biomolecules (adenine (A), thymine (T), cytosine (C), guanine (G) from DNA, and β-carotene) and water pollutants (malachite green) is also demonstrated here.

## Methods

### Fabrication of AgNP/AAO Substrate

A glass slide with 150 nm of aluminum (Al) thin film deposited by sputtering was anodized in the sulfuric acid (0.3 M) using a voltage of 16 V to form porous AAO substrates with arrays of self-organized nanochannels with the specific pore diameters and interparticle gaps. The AAO nanochannels were then chemically etched in phosphoric acid and chromic acid at 35 °C to achieve optimal pore size and interparticle gap for this study. To grow Ag nanoparticles in the AAO nanochannels by electrochemical plating, an alternating voltage of 9 V was applied to the AAO substrate in a solution of silver nitrate (0.006 M) and magnesium sulfate (0.165 M) (molarity ratio: 1: 27.5) for different time duration. After depositing Ag nanoparticles to the desired length, the substrate was washed by hydrochloric acid (0.2 M) and formed the final AgNP/AAO SERS-active substrate [12].

### SERS Measurements of Biomolecules and Water Pollutant

DNA canonical nucleobases (adenine, thymine, cytosine, and guanine), β-carotene, and malachite green, used as model pollutants in water, were purchased from Sigma-Aldrich. They were all used without further purification. Sample solutions were prepared by dissolving in water at designated concentrations. Five microliters of each sample solution was dropped on the surface of the AgNP/AAO substrate and dried for 20 min before the Raman measurements [13]. The morphology of AgNP/AAO substrates was performed on a field-emission scanning electronic (SEM) microscope (FESEM, JSM-6700F, JEOL) and atomic force microscope (AFM) (Dimension 3100, Bruker) in tapping mode.

Raman measurements were carried out in a commercial Raman microscope (HR800, Horiba) with a He–Ne laser (632.8 nm) as the excitation light source. The laser beam, after passing through a laser-line filter to remove residual plasma lines, was focused by an objective lens to the substrate surface. In this experiment, ×20 objective lens (spot size is about 20–30 μm) was used to evaluate the uniformity and reproducibility of SERS signal. The scattering radiation was collected by the same objective lens and sent to an 80-cm spectrometer (1800 gr/mm) plus liquid-nitrogen-cooled charge-coupled device for spectral recording. The resultant spectral resolution and accuracy are 3 and 0.1 cm^−1^, respectively. The irradiated laser power was adjusted to prevent any laser-induced damage such that the portrayed Raman signal is linearly related to the laser power. The signal acquisition time was 60 s. Each acquired SERS spectrum was processed with a home-made software to remove high-frequency noise and continuum background [13].

## Results and Discussion

### Characterization of AgNP/AAO Substrate

**Fig. 1:**
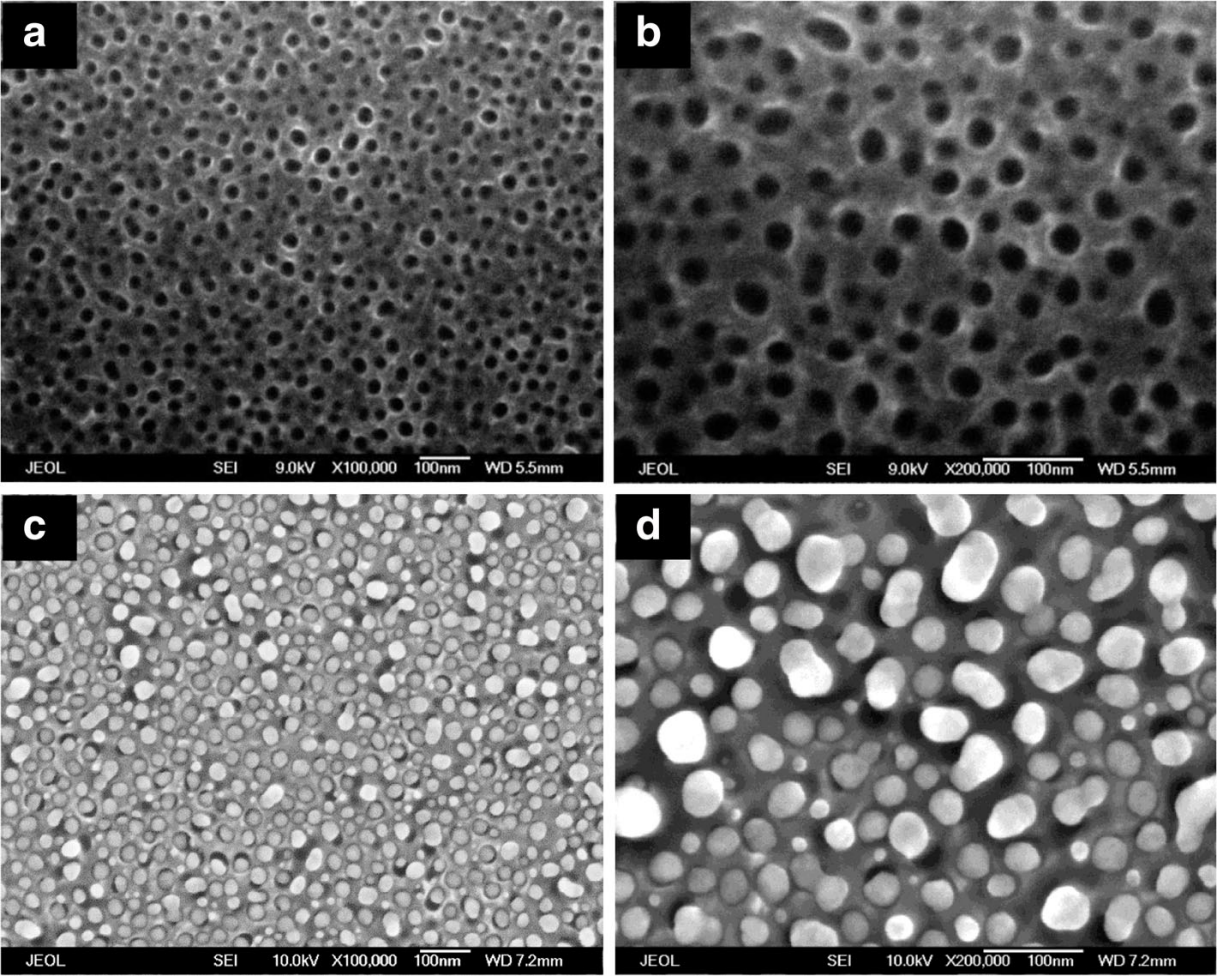
Top-view scanning electron microscope images. **a**, **b** Anodic aluminum oxide (AAO) template. **c**, **d** Ag nanoparticle array grown in the AAO template. **a, c** magnification 100 K. **b, d** magnification 200 K

**Fig. 2:**
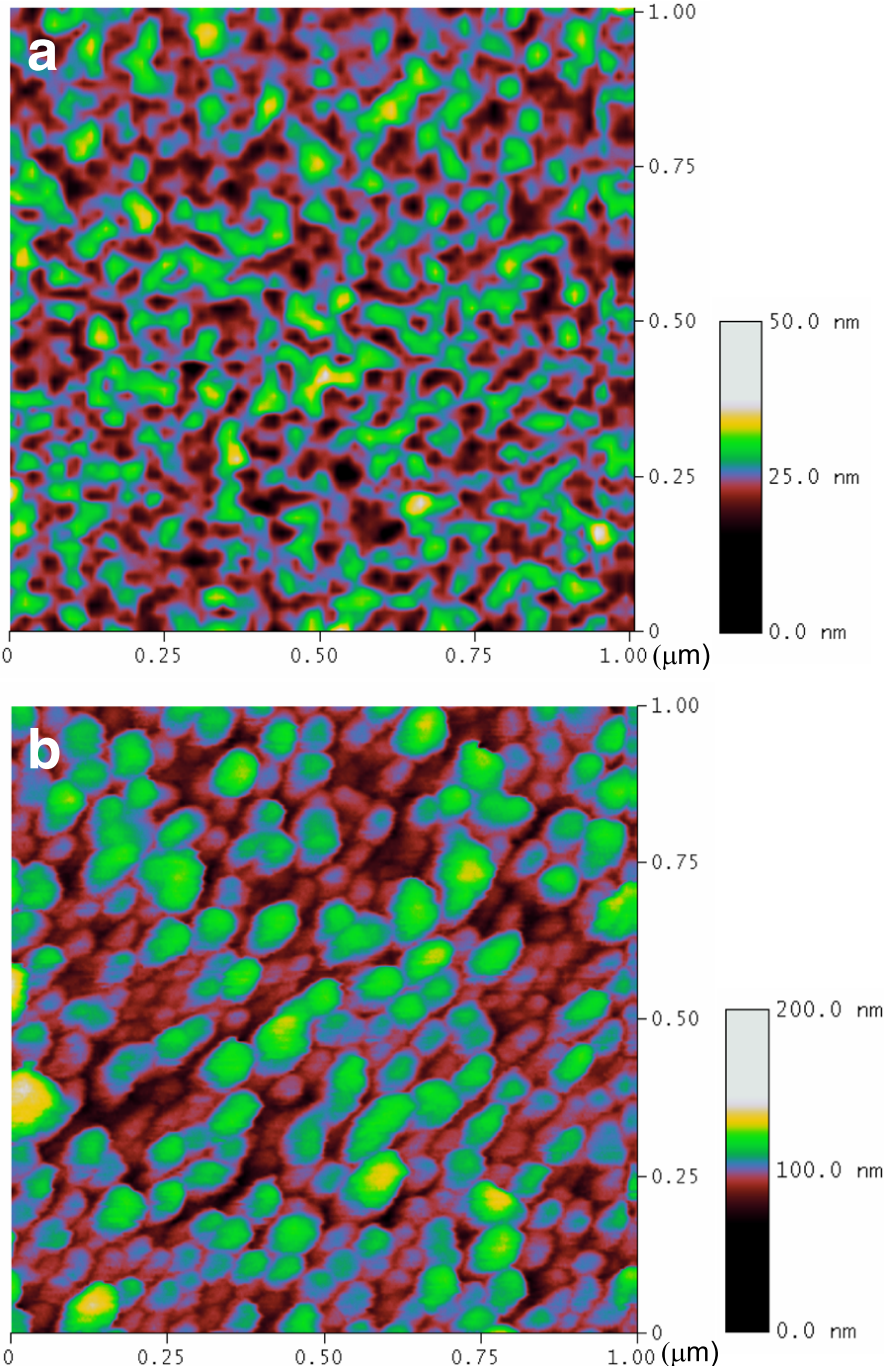
Topographic images obtained with atomic force microscope (AFM). **a** AAO template. **b** Ag nanoparticles array grown in AAO template

Figure 1 shows top-view SEM images of AAO nanochannels and AgNP/AAO substrate. The pore diameter of AAO channels is about 20–40 nm, and the inter-channel gap is about 10–20 nm, as shown in Fig. 1a, b. The Ag nanoparticles have diameters ranging from 20 to 50 nm, and the interparticle spacing ranging from 10 to 30 nm, as revealed in Fig. 1c, d. Figure 2 shows the topography of a typical AAO nanochannel (Fig. 2a) and AgNP/AAO substrate (Fig. 2b) revealed by AFM measurements. The average roughness of AAO nanochannels is about 3.38 nm (RMS) and 2.74 nm (Ra), and their pore size is about 20 ± 8 nm using bearing analysis of AFM software (Nanoscope). These numbers are similar with that obtained from SEM images. On the other hand, the diameter of Ag nanoparticles is about 33 ± 10 nm and the roughness of AgNP/AAO substrate is about 11.78 nm (RMS) and 9.76 nm (Ra). The larger diameter probably resulted from the overgrowth of Ag nanoparticles above the top surface of the AAO nanochannels, while the larger roughness values may be caused by different growth speeds of Ag nanoparticles [12].

### Performances of SERS Substrate: Reproducibility and Enhancement

**Fig. 3:**
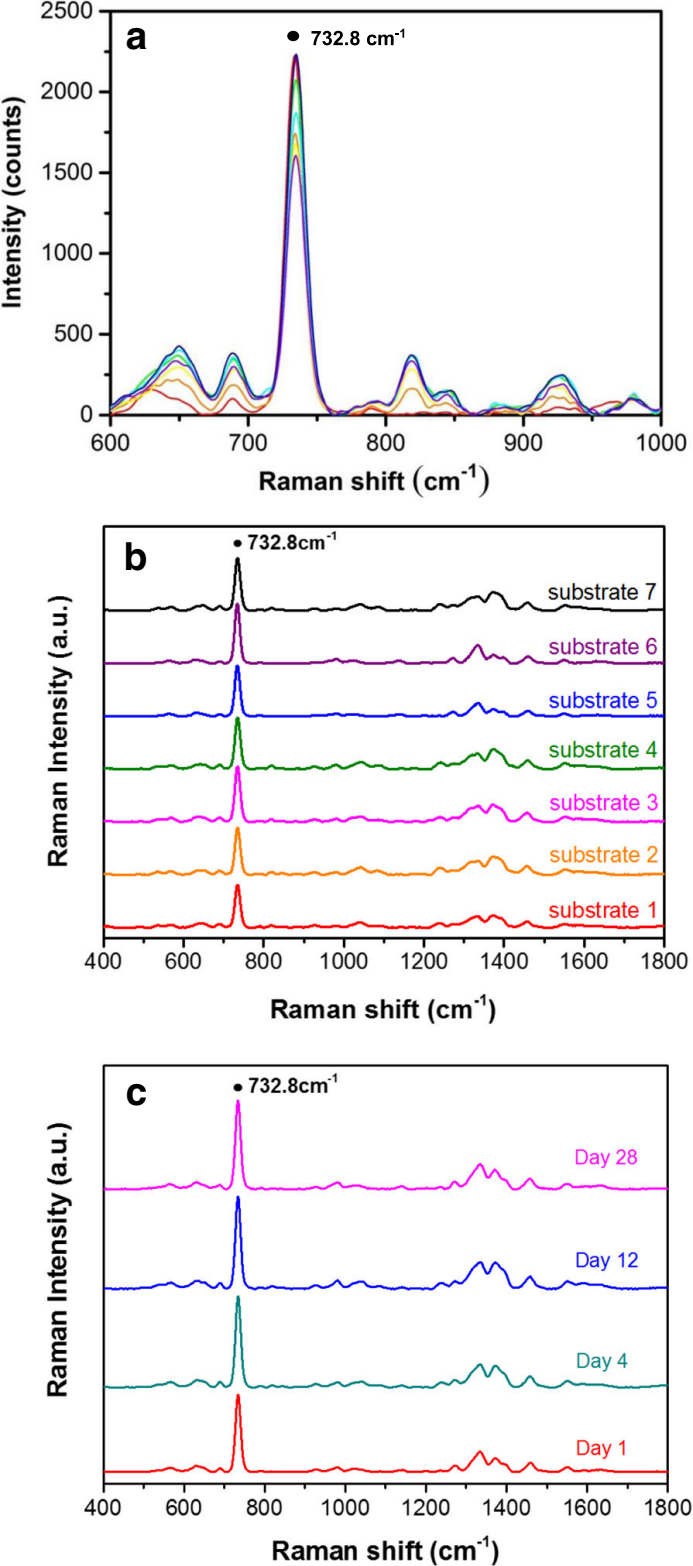
The uniformity, reproducibility, and durability tests of AgNP/AAO surface-enhanced Raman scattering (SERS) substrate. **a** SERS spectra of adenine (10^−4^ M) obtained at seven spots separated by about 10–15 mm on the SERS substrate. **b** SERS spectra of adenine (10^−4^ M) obtained at seven different SERS substrates. **c** SERS spectra of adenine (10^−4^ M) obtained at delays of 1, 4, 12, and 28 days after production

**Fig. 4:**
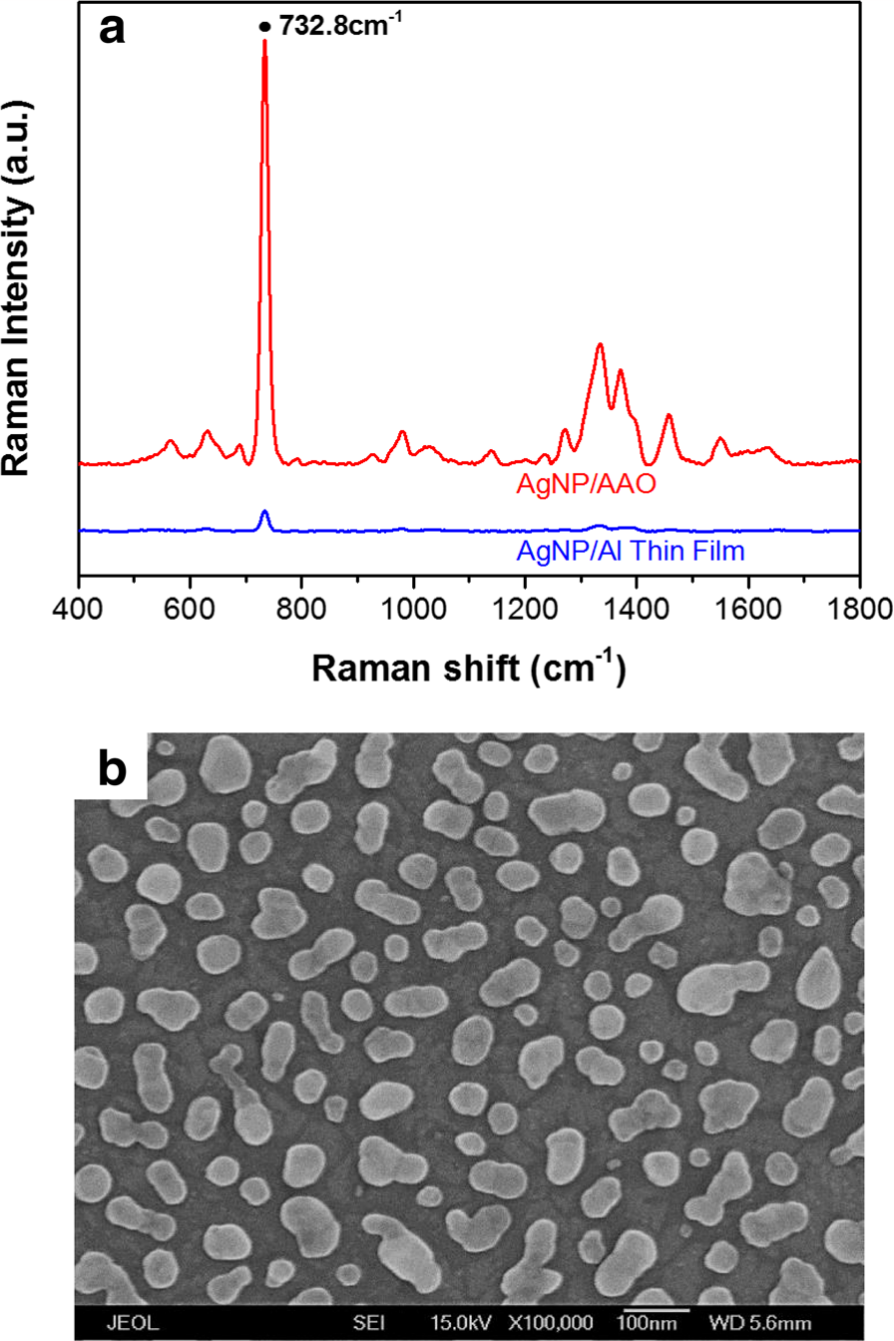
**a** SERS spectra of adenine (10^−4^ M) on AgNP/AAO substrate and Ag nanoparticles electrochemically plated on the aluminum (Al) thin film (AgNP/Al) substrate (without AAO template) by averaging seven spectra taken at different spots of the substrate, respectively. **b** Top-view scanning electron microscope image of AgNP/glass substrate

In consideration of SERS reproducibility, three performance evaluations were made. Firstly, what does the SERS enhancement vary across the substrate? Figure 3a shows SERS spectra of adenine (10^−4^ M) at seven different spots separated by about 10–15 mm on AgNP/AAO substrate. The SERS spectra of adenine are similar with our previous works [12–20]. The uniformity of signal strength at the prominent 732.8-cm^−1^ peak varies about 10–20%. Secondly, what does the SERS enhancement differ from substrate to substrate? Figure 3b shows SERS spectra of adenine (10^−4^ M) on different AgNP/AAO substrates. The reproducibility result shows that the SERS signal at 732.8 cm^−1^ is varied by about 10% at seven different AgNP/AAO substrates, by averaging seven spectra taken at different spots of the substrate, respectively. Lastly, what does the SERS enhancement alter in time? Figure 3c shows SERS spectra of adenine (10^−4^ M) obtained at delays of 1, 4, 12, and 28 days after production. The durability result shows that the SERS signal at 732.8 cm^−1^ is varied by about 8% in almost 1 month, indicating very high durability of AgNP/AAO substrate. The other performance issue is how the enhancement of AgNP/AAO substrate is compared with other SERS substrates. Figure 4a shows the comparison of SERS spectra of adenine (10^−4^ M) acquired on AgNP/AAO substrate and Ag nanoparticles electrochemically plated on the aluminum (Al) thin film (AgNP/Al) substrate (without AAO templates). For this special case, the SERS sensitivity of AgNP/AAO substrate is about 17 times of that of AgNP/Al substrate. Figure 4b shows top-view SEM image of AgNP/Al substrate.

Surface enhancement factors (EFs) were calculated by the following formula in our previous works [15, 23, 24].

1 $$ \mathrm{E}\mathrm{F} = \frac{I_{\mathrm{SERS}}/{N}_{\mathrm{SERS}}}{I_{\mathrm{Raman}}/{N}_{\mathrm{bulk}}} $$

where *N*_bulk_ is the number of analyte molecules (adenine) sampled in the bulk, and *N*_SERS_ is the number of adenine adsorbed on the SERS substrates. *I*_SERS_ and *I*_Raman_ denote the integrated intensities at specific peaks (732.8 cm^−1^) in the SERS and Raman spectra. With the same spot size of the laser and the same content of adenine, the ratio of *N*_SERS_ to *N*_bulk_ could be deemed as the ratio of two concentrations of adenine. The EF value of SERS substrate with and without the AAO template are 1.9 × 10^8^ and 1.1 × 10^7^, respectively. The result shows that the particle size and interparticle gap of Ag nanoparticles can be effectively manipulated by AAO templets to enhance the SERS sensitivity. From the literatures [25–29], the EF of the Ag-based SERS substrate is in the broad range of 10^2^~10^9^, which depends on the density and layers of Ag nanoparticles, molecule adsorbability on the SERS substrate, and the parameter setting of Raman spectroscopy (e.g., different wavelengths of the laser). In other words, the higher EF would induce the poorer reproducibility. For the one layer of Ag nanoparticles system, our SERS substrate exhibits enough enhancement factor (>10^8^) with great reproducibility (~10%) from substrate to substrate.

By the way, although it is common to compare the enhancement factor among different SERS enhancers, it is not the only performance factor that is relevant to the applications of SERS technology. The following factors are equally important, if not more: reliability, uniformity, ease of operation, large size, speed, etc. Our Ag/AAO SERS substrate in this work exhibits these advantages, compared with that of other Ag systems.

### SERS Detection of Canonical Nucleobases of DNA

**Fig. 5:**
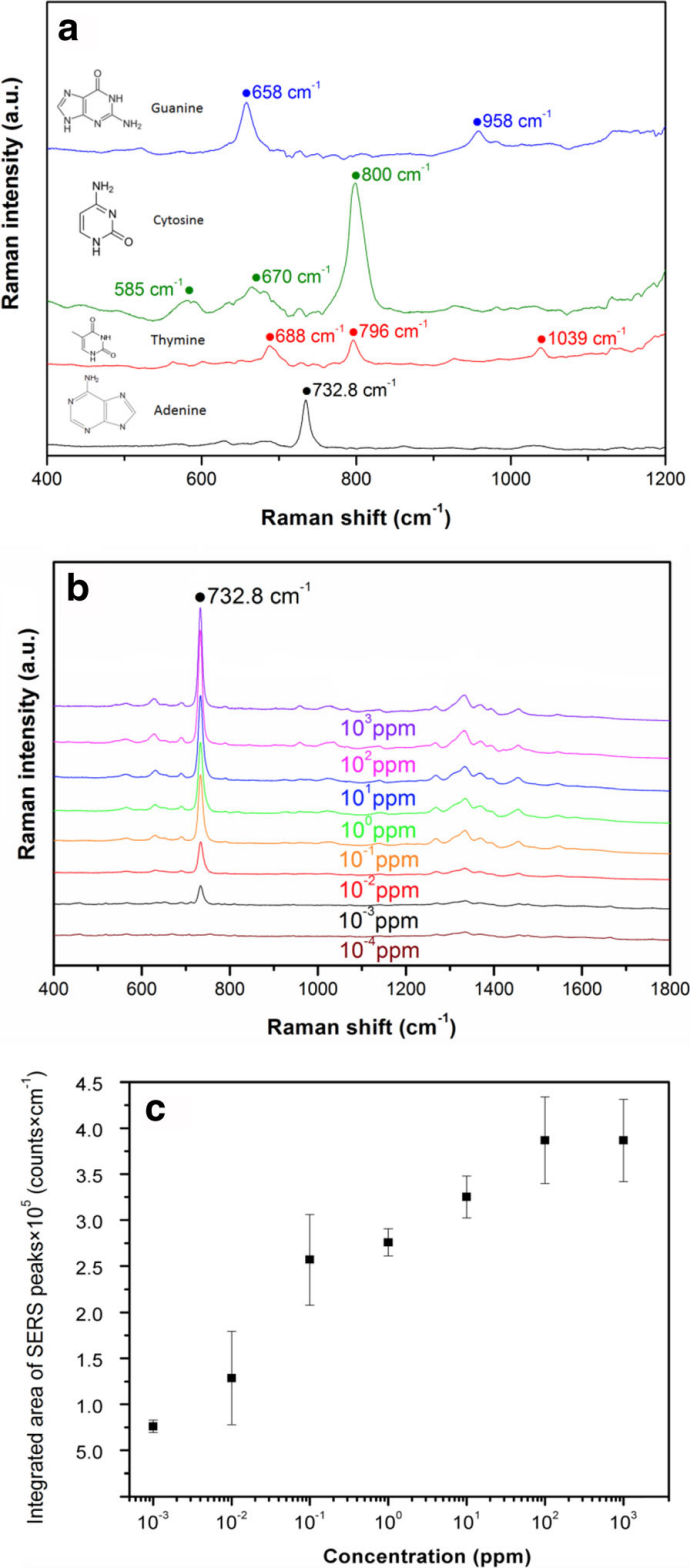
**a** SERS spectra of adenine, thymine, cytosine, and guanine (10^−4^ M). **b** SERS spectra of adenine at different concentrations (from 10^−4^ to 10^4^ ppm). **c** Integrated area of 732.8 cm^−1^ peak of adenine (${I}_{732.8}^{*}$) as a function of concentration

In SERS detection, 5 μL of the small biomolecules (10^−4^ M), adenine (A), thymine (T), cytosine (C), guanine (G) from canonical nucleobases of DNA, were dropped on the Ag–AAO nanoparticle arrays. SERS spectra in the range of 400 to 1200 cm^−1^ were observed for the small biomolecules. The results in Fig. 5a show that the characteristic peaks of four biomolecules can be clearly differentiated by SERS detection platform. A significant SERS peak (732.8 cm^−1^) was observed in the adenine (A), which is similar with our previous results [15–20]. SERS peaks of thymine (T) were found at 688, 796, and 1039 cm^−1^. SERS peaks of cytosine (C) and guanine (G) were measured at 585, 670, 800, 658, and 958 cm^−1^, respectively. Furthermore, the calibration curves of adenine were observed and calculated in Fig. 5b, c. The integrated peak area (732.8 cm^−1^) increases with the logarithm concentration of adenine (0.001–100 ppm) increase, and the limit of detection (LOD) concentration of adenine is lower than 0.001 ppm, as shown in Fig. 5b, c. These SERS peaks of DNA canonical nucleobases are rapidly, precisely, label-freely detected within 10 min, which might be potential for rapid detection of DNA or bacteria in the future.

### SERS Detection of Water Pollutant (Malachite Green)

**Fig. 6:**
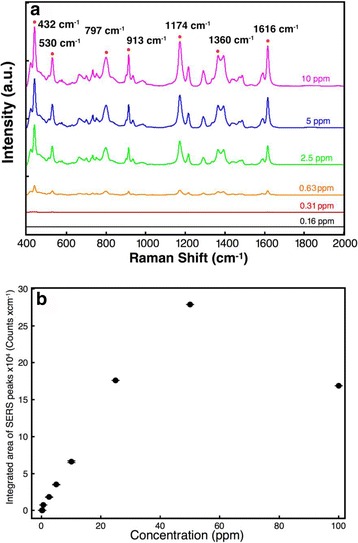
**a** SERS spectra of malachite green at different concentrations (from 0.16 to 100 ppm). **b** Integrated area of 1174 cm^−1^ peak of adenine (${I}_{1174}^{*}$) as a function of concentration

Malachite green has been popularly applied in the cultivation industry. It can stay in eatable fish tissues for a long time, which would induce carcinogenesis and teratogenesis [30–32]. The investigation of Ag–AAO nanoparticle arrays in situ detection of biocide (malachite green) in water was evaluated in Fig. 6a. It shows the outstanding resolution of SERS spectra of malachite green in the difference concentrations (0.16–100 ppm). The strongest SERS peak was found at 1174 cm^−1^, and other peaks were observed at 432, 530, 797, 913, 1360, and 1616 cm^−1^, respectively. The result shows that these peaks are obviously differentiated by Ag–AAO nanoparticle and display excellent sensitivity. Water solution containing 0.63–50 ppm of malachite green can be readily detected by SERS based on substrates of Ag–AAO nanoparticle arrays, exhibiting linear-like relationship between SERS intensity and concentrations, Fig. 6b. The LOD of malachite green was found to be 0.63 ppm, which is sensitive enough to detect the biocide in the water. Compared with malachite green of SERS detection in the literature [29], the LOD of malachite green by SERS detection is about 10^−6^ M, which is similar with our result (LOD <0.63 ppm (<1.7 × 10^−6^ M)). Therefore, the extreme dilute solution of water pollutants, such as biocides and pesticides, can be monitored by this SERS platform technology.

### SERS Detection of β-Carotene

**Fig. 7:**
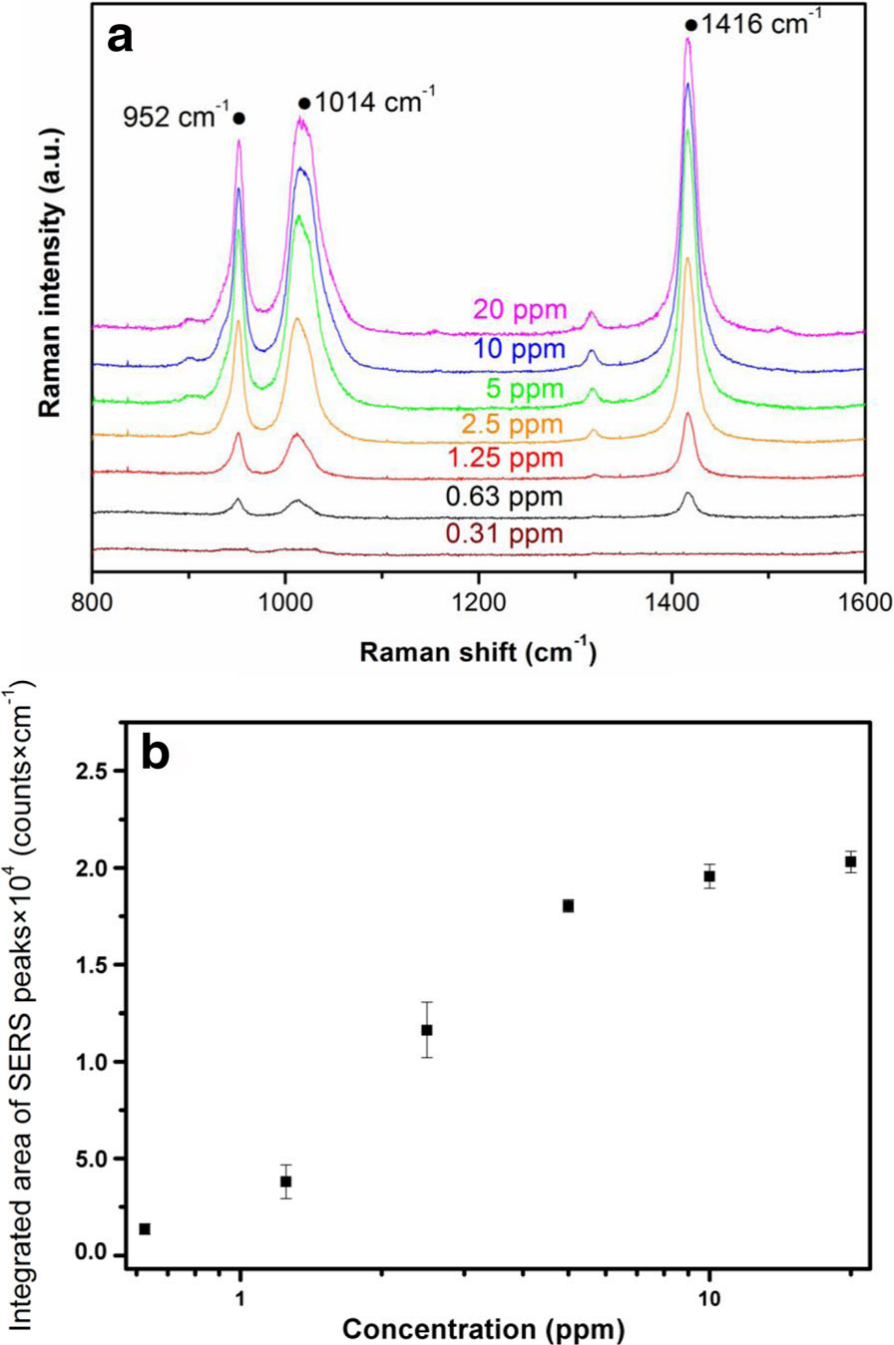
**a** SERS spectra of β-carotene at different concentrations (from 0.31 to 20 ppm). **b** Integrated area of 1416 cm^−1^ peak of adenine (${I}_{1416}^{*}$) as a function of concentration

β-carotene plays an important role of antioxidant defense system in the skin, which can be a substance that inhibits the oxidation of other biomolecules. It can protect the body from free radicals and therefore be considered as an indicator for the health index. It induces skin malignancies and cancer by the disruptive effects of free radicals and reactive oxygen species, if antioxidants are unbalanced [33]. SERS spectra of small biomolecules (β-carotene) with various concentrations (0.31–20 ppm) were investigated in Fig. 7a. Three sharp SERS peaks were observed at 952, 1014, and 1416 cm^−1^. The integrated area of the three peaks increases with the concentration of β-carotene (0.63–5 ppm) increase, but arrives at saturation at 10–20 ppm, Fig. 7b. The higher concentration (10–20 ppm) of β-carotene causes the laser randomly scattering to interfere in SERS detection. The LOD of β-carotene is lower than 0.63 ppm. Furthermore, a great interest has recently been focused on the detection of lycopene and β-carotene due to its preventive activity against several pathologies, such as cardiovascular disease and some cancer types, such as prostate, gastrointestinal, and epithelial. In the literature [34], the LOD obtained for UV–vis detection-HPLC method is 3 × 10^−4^ M for β-carotene and 6 × 10^−4^ M for lycopene. However, the LOD of β-carotene by our SERS detection is <0.63 ppm (<1.2 × 10^−6^ M), which is much higher than the UV–vis detection-HPLC method. Therefore, the high sensitivity SERS detection of β-carotene can be useful for the rapid sensing antioxidant capability to exhibit the health index for the healthcare.

In our knowledge, compared with other Ag systems, Ag/AAO SERS substrate could detect a variety of small biomolecules (adenine (A), thymine (T), cytosine (C), guanine (G), β-carotene) and water pollutants (malachite green), which can be used extensively in different fields without further labelling or modification.

## Conclusions

This paper demonstrates that Ag–AAO nanoparticle arrays can be reproducibly fabricated and exploited for SERS-based detection of small biomolecules such as canonical nucleobases of DNA, β-carotene, and water pollutants. The uniformity of SERS signal of adenine varies about 10–20% at seven different spots on AgNP/AAO substrate. The reproducibility result shows that SERS signal of adenine is varied by about 10% at seven different AgNP/AAO substrates. Furthermore, the durability result shows that the signal strength of adenine varied by 8% even after 28 days. The high sensitivity, uniformity, and reproducibility of this SERS-active substrates based on AgNP/AAO can in principle be used for the quantitative determination of the label-free health diagnostics, bio-sensing, and water detection, such as sensing antioxidant capability (β-carotene), detecting DNA canonical nucleobases (adenine, thymine, cytosine, guanine), and in situ monitoring water pollutants (malachite green), respectively.
